# Preventing malnutrition within the first 1000 days of life in under-resourced communities: An integrative literature review

**DOI:** 10.1177/13674935231166427

**Published:** 2023-04-03

**Authors:** Marian Joyce Nyarko, Dalena (RM) van Rooyen, Wilma ten Ham-Baloyi

**Affiliations:** 1 Faculty of Health Sciences, 56723 Nelson Mandela University, Port Elizabeth, South Africa

**Keywords:** breast feeding, complementary feeding, developing countries, infancy or early childhood, prevention of malnutrition, review

## Abstract

This integrative review aimed to summarise existing best evidence practice for preventing malnutrition within the First 1000 Days of Life in under-resourced communities. BioMed Central, EBSCOHOST (Academic Search Complete, CINAHL and MEDLINE), Cochrane Library, JSTOR, Science Direct and Scopus were searched as well as Google Scholar and relevant websites for grey literature. Most recent versions of strategies, guidelines, interventions and policies; published in English, focussing on preventing malnutrition in pregnant women and in children less than 2 years old in under-resourced communities, from January 2015 to November 2021 were searched for. Initial searches yielded 119 citations of which 19 studies met inclusion criteria. Johns Hopkins Nursing Evidenced-Based Practice Evidence Rating Scales for appraising research evidence and non-research evidence were used. Extracted data were synthesised using thematic data analysis. Five themes were derived from extracted data: 1. Improving social determinants of health using a multisector approach; 2. Enhancing infant and toddler feeding; 3. Managing healthy nutrition and lifestyle choices in pregnancy; 4. Improving personal and environmental health practices; and 5. Reducing low-birthweight incidence. Further exploration regarding preventing malnutrition in the First 1000 Days in under-resourced communities is required using high-quality studies. Systematic review registration number: H18-HEA-NUR-001 (Nelson Mandela University).

## Background

The First 1000 Days of a child’s life, from the day of conception until the child is 2 years old, have been described as a crucial period when poor nutrition will have a long-lasting impact on the future growth and development of that child (Cusick and Georgieff, 2016). During this period, adequate nutrition is vital for the foetus and child’s growth and development (Black et al., 2013). This period is therefore dubbed ‘the 1000-days window of opportunity’, as correct nutrition and care of the unborn baby, through the pregnant woman, and of children under 2 years have a long-lasting effect on a family’s future health and economic success (Lowensohn et al., 2016; Ritte et al., 2016; USAID, 2015; WHO, 2013).

Malnutrition, which is (Sizer and Whitney, 2017:3) defined as a condition caused by excess or deficiency in food nutrients or energy intake or by an imbalance of nutrients, when occurring in the First 1000 Days of Life, can have a detrimental effect. Such effects can be on the child’s growth (leading to stunting – lower height for age), cognitive development (low intelligence quotient and attention deficits) and associated school performance, and on immune function, including developing non-communicable diseases in adulthood or conditions leading to death (Dang et al., 2013; Desyibelew and Dadi, 2019; Prado and Dewey, 2014; Sizer and Whitney, 2017; Smith, 2016).

Household food insecurity due to a lack of adequate family income most severely affects pregnant women, breastfeeding mothers, babies and children (Boyle, 2017; Ramsey et al., 2011). A woman’s socio-economic circumstances, partner’s educational level and area of residence may increase the risk of her and or her child becoming malnourished in pregnancy (Boch et al., 2021; Desyibelew and Dadi, 2019).

With regards to children, inadequate childcare, single motherhood, maternal illness and low family income increase malnourishment risk (Tette et al., 2016). Hunger is costly as it ultimately affects individuals’ health status, reduces economic productivity and increases the chances of individuals going to live in under-resourced communities and becoming caught in an inter-generational cycle of poverty and malnutrition (Boyle, 2017; Watkins, 2016).

The United Nations’ 17 Sustainable Development Goals, which serve as a roadmap for eradicating poverty and hunger whilst improving health and welfare of populations and simultaneously protecting the planet, support the notion that nutrition impacts on every aspect of life (Grosso et al., 2020). Malnutrition in the First 1000 Days of Life must therefore be prevented to avoid irreversible health problems in childhood and adulthood, especially in under-resourced communities (Martorell, 2017). Under-resourced communities are those that experience high rates of unemployment and poor access to housing, safe potable water, sewage and sanitation, clean environment and food security, thereby increasing risks of repeated infections, which may lead to severe malnutrition (Lehohla, 2013). Although some reviews have been conducted on preventing malnutrition, these focussed predominantly on hospitalised patients (Collins and Porter, 2015), or included pregnant women in Africa only (Desyibelew and Dadi, 2019). Summarising best evidence on preventing malnutrition in the First 1000 Days of Life in under-resourced communities will allow for consolidation of research evidence in this context, identification of research gaps and assist in developing best practice guidelines or interventions to prevent malnutrition for these populations.

### Aim

To summarise existing best evidence for preventing malnutrition within the First 1000 Days of Life in under-resourced communities.

## Methods

Whittemore and Knafl’s (2005) five-stage approach to an integrative literature review was used as follows: Stage 1: Formulation of the review question; Stage 2: Search and collection of literature; Stage 3: Critical appraisal of collected literature; Stage 4: Data analysis; and Stage 5: Presentation of data. The first author conducted this review, under supervision of the second and third authors.

This integrative literature review was part of a larger, doctoral study aimed at developing strategies preventing malnutrition in the First 1000 Days of Life in under-resourced communities. The review was guided by the following question: What evidence is available regarding preventing malnutrition within the First 1000 Days of Life in under-resourced communities?

### Literature search

Searching literature for evidence was managed by the first author with assistance of a librarian. Electronic databases searched included BioMed Central, EBSCOHOST (Academic Search Complete, CINAHL and MEDLINE), Cochrane Library, JSTOR, Science Direct and Scopus. Grey literature was identified using supplemental search methods, Google Scholar, and relevant websites, including United Nations International Children's Emergency Fund (UNICEF)’s website. Hand searching reference lists of included articles and articles submitted by Mendeley to the researcher’s electronic mailbox was also conducted.

The keywords used included: ‘Infancy or early childhood’ AND ‘prevention of malnutrition’ AND ‘developing countries’ OR ‘developing nations’ OR ‘third world’ OR ‘low income countries’. Occasionally, a keyword had to be substituted with a synonym to yield relevant results.

Literature was included from all five levels of evidence, as defined by Johns Hopkins Nursing Evidence-Based Practice (JHNEBP) (Newhouse et al., 2007), and included strategies, guidelines, interventions and policies published in English. Selected literature focussed on preventing and addressing malnutrition in pregnant women and in children less than 2 years of age in under-resourced communities, published between January 2015 to November 2021 to obtain the most recent information. Only most recent updates of a relevant strategy, guideline, or policy were used. Strategies, guidelines, interventions and policies developed for well-resourced communities and study protocols were excluded.

Titles and abstracts were read, and full texts of literature deemed relevant was obtained. Full texts were read and selected, based on inclusion and exclusion criteria.

### Critical appraisal

The first author and an independent reviewer critically appraised the articles using the JHNEBP model and guidelines’ Evidence Rating Scale for appraising research evidence and non-research evidence (Newhouse et al., 2007). Research evidence was scored by assessing how many of the 12 elements for research evidence and 11 for non-research evidence were adhered to, with results being presented as percentages to show uniformity of scores presentation. Each element that was adhered to, scored 1. Total obtained scores were divided by the total score (11 or 12) times 100. Only scores of 66% and above were considered to have sufficient methodological rigour to be included in this review.

Critical appraisal was conducted by the first author and the critical reviewer independently, who reached consensus on inclusion and exclusion of articles.

### Data extraction and synthesis

Data related to the reference of each article, design, strength and quality of evidence and recommendations regarding prevention of malnutrition in the First 1000 Days was extracted from included articles, using an adaptation of JHNEBP individual summary sheet. Extracted data were synthesised using thematic data analysis. Data were presented through narratives.

### Ethical considerations

Ethical clearance was obtained from xxx University (ethics number: xxx). Avoiding plagiarism, whilst honestly and transparently portraying extracted data, were maintained throughout.

## Results

### Search outcome

**Figure 1.**
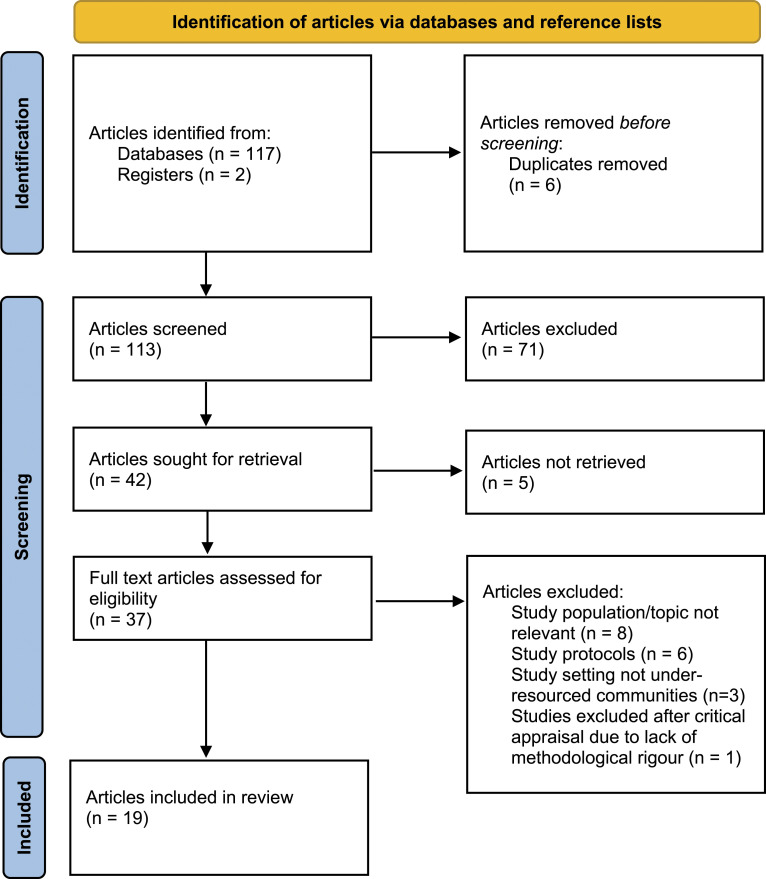
PRISMA flow chart of the search and selection process of articles (adapted from Page et al., 2021).

A total of 119 articles were identified. Six duplicated articles were excluded. Of 113 articles remaining, 71 irrelevant articles were excluded after screening titles and abstracts, including five full text articles that were unobtainable. Thirty-seven full articles were then read. Seventeen (eight with population/topics that were not relevant, six protocols and three settings that were not under-resourced) as well as one article after critical appraisal due to lack of methodological rigour (scoring less than 66%) were then excluded. Nineteen articles were included in the review (see Figure 1).

### Levels of evidence

Out of 19 articles included, there was a meta-analysis of randomised control trials combined with a systematic review as well as a randomized controlled trial (Level I) (*n* = 2; 10.5%). Another was a quasi-experimental study (Level II) (*n* = 1; 5.3%), ten (*n* = 10, 20.5%) were non-experimental studies (Level III), five (*n* = 5; 26.3%) were internationally recognised expert opinions (Level IV) and one was a clinical practice guideline (Level V). Majority of included research articles related to research conducted in African countries (*n* = 11, 57.9%), while other articles included two studies (*n* = 2; 10.5%) from South Asia. Six studies (*n* = 6; 31.6%) entailed international research including five expert opinion papers and one systematic review.

### Themes

Five themes emerged from synthesising 19 included articles. These were: 1. Improving social determinants of health using a multisector approach (*n* = 14; 73.7%); 2. Enhancing infant and toddler feeding (*n* = 14; 73.7%); 3. Managing healthy nutrition and lifestyle choices in pregnancy (*n* = 10; 52.6%); 4. Improving personal and environmental health practices (*n* = 8; 42.1%); and 5. Reducing low-birthweight incidence (*n* = 7; 36.8%). Five articles addressed all five themes, one discussed four themes, five articles addressed three themes, a further two addressed two themes and six addressed only one theme (see Table 1).

**Table 1.** Themes per included article and topics covered by article.

Articles	Improving social determinants of health using multi-sector approach	Enhancing infant and toddler feeding	Managing healthy nutrition and lifestyle choices in pregnancy	Improving personal and environmental hygiene practices	Reducing low-birthweight incidence	Topics covered per article
Agho et al. (2019)	√	√	√	√	—	**4**
Akombi et al. (2017)	√	√	√	√	√	**5**
Bhutta et al. (2017)	√	√	√	—	—	**3**
Black et al. (2017)	√	—	—	—	—	**1**
Britto et al. (2017)	√	√	√	√	√	**5**
Carducci and Bhutta (2018)	√	√	√	√	√	**5**
De Vita et al. (2019)	√	√	√	√	√	**5**
Du Plessis et al. (2018)	√	√	√	—	—	**3**
Hossain et al. (2020)	√	—	—	—	—	**1**
Mohammed et al. (2019)	√	√	√	√	√	**5**
Picolo et al. (2019)	—	√	—	—	—	**1**
Quelhas et al. (2018)	—	-	√	—	√	**2**
Raymond et al. (2017)	√	√	—	—	√	**3**
Richter et al. (2017)	√	√	—	√	—	**3**
Rose et al. (2015)	√	√	—	√	—	**3**
Shankar et al. (2019)	—	—	√	—	—	**1**
Tebeje et al. (2019)	√	√	—	—	—	**2**
Van der Kam et al. (2016)	—	√	—	—	—	**1**
Zerfu and Biadgilign (2018)	—	—	√	—	—	**1**
Total	14	14	11	8	7	**Total**

Synthesised data will now be described under five themes, referring to relevant articles under each theme.

### Improving social determinants of health using a multisector approach

Improving social determinants of health, using a multisector approach was discussed by 14 out of 19 articles as being necessary for preventing malnutrition in the First 1000 Days of Life within under-resourced communities. Living in unhygienic conditions, not being vaccinated, food insecurity, poverty, poor child feeding practices, unhygienic practices and inadequate healthcare may result in malnutrition and should be considered when designing strategies for preventing malnutrition (Black et al., 2017; De Vita et al., 2019; Mohammed et al., 2019; Raymond et al., 2017; Tebeje et al., 2019).

Evidence-based integrated multi-sector or multi-dimensional interventions or approaches were recommended, preferably starting from adolescent health and continuing through pregnancy and childcare (Richter et al., 2017). Britto et al. (2017) recommended preconception care, antenatal care, care during labour and delivery and childcare, as well as responsive parenting, maternal wellbeing, social protection and water, sanitation and hygiene (WASH – being a key public health issue and part of targets within WHO’s Sustainable Development Goal 6), to improve cognitive and physical development of children, including unborn children. An integrated approach is needed to ensure that health, nutrition, cognitive stimulation, parenting, social protection, safety, and access to safe water and hygiene are available to enhance child development, and for strengthening family support, protection and nurturing care (Britto et al., 2017).

Specific recommendations of Rose et al. (2015) as well as Bhutta et al. (2017) included breastfeeding, increased immunisation, vitamin A supplementation, hand washing, poverty alleviation and food insecurity to prevent and reduce malnutrition in children.

Carducci and Bhutta (2018) discussed preventing small for gestational age (birthweights below the 10th percentile for babies of similar gestational age) and low-birth weight babies (those weighing less than 2.5 kg at birth) by intensifying the provision of social interventions and care before pregnancy and in pregnancy, as malnutrition risks are higher in such babies. Like Carducci and Bhutta, 2017 advocated for improving sanitation and maternal nutrition to decrease low-birth weight at an individual level in Nigeria. Community interventions should include poverty alleviation interventions, targeting uneducated mothers living in under-resourced communities (Akombi et al., 2017). Similar recommendations were also made by Hossain et al. (2020):1). In a study conducted in disadvantaged districts from Rwanda, Tanzania and Uganda, recommendations were that health policies to address childhood malnutrition should focus on poor households, improving sanitation and education of mothers to attend regular immunisation and growth monitoring sessions (Agho et al., 2019). A similar recommendation was made by Bhutta et al. (2017).

Finally, nutrition strategies for the First 1000 Days of Life should be developed that involves all stakeholders, including Departments of Health, Social Development and leaders from under-resourced communities and its implementation must be driven by nutrition champions, identified from stakeholders involved (Du Plessis et al., 2019:9).

### Enhancing infant and toddler feeding

Enhancing infant and toddler feeding was mentioned in 14 of 19 articles reviewed. Infant and toddler feeding are discussed as feeding during the first 6 months of life and feeding from 6 months onwards.

#### Infant feeding during the first 6 months

Breastfeeding for the first 6 months was recommended as it was found to be protective against undernutrition (Rose et al., 2015). As breastmilk has numerous benefits for both child and mother, breastfeeding should be promoted, supported and protected, as recommended by WHO and UNICEF when implementing the Baby-Friendly-Hospital-Initiative (aiming at implementing practices that protect, promote and support breastfeeding) and should be seen as the main food for infants to reduce childhood illnesses and deaths due to how suboptimal infant feeding affects child immunity (Agho et al., 2019; Carducci and Bhutta, 2018; Richter et al., 2017).

Breastfeeding duration has been found to correlate with children’s intelligence, so mothers should be supported to initiate breastfeeding at birth, through implementation of Baby-Friendly-Hospital-Initiatives, as it increases chances of mothers breastfeeding for longer, reducing risks for malnutrition (Britto et al., 2017). For growth-restricted infants, Carducci and Bhutta (2018) recommend kangaroo mother care (KMC). With KMC, the baby is in continuous skin-to-skin contact with the mother, who breastfeeds it frequently. KMC practice has been found to reduce hypothermia, hypoglycaemia and morbidity as well as promote a faster rate of physical growth when compared with babies without KMC (Carducci and Bhutta, 2018). Mothers who breastfeed for longer were found to take special effort to ensure their infants get nutritious complementary food, probably because they are better informed (Tebeje et al., 2019).

Using trained community volunteers to support mothers to exclusively breastfeed for 6 months and to show mothers how to prepare nutritious food for their children were interventions recommended by (Rose et al., 2015:9). Information on hygienic practices and childhood illnesses through quality health services must also be provided to prevent malnutrition (Mohammed et al., 2019:2).

#### Feeding after 6 months

Exclusive breastfeeding for 6 months and continuing breastfeeding for 2 years, with appropriate complementary feeding, reduce malnutrition risks in children (Bhutta et al., 2017; Tebeje et al., 2019). At 6 months, milk alone does not provide required nutrients that an infant need, so other foods must be added to prevent malnutrition. Introduction of foods either than milk should be timely (at 6 months), be varied (from different food groups), be adequate (age-appropriate) and given at right frequencies (2–3 times daily for infants 6–8 months and 3–4 times daily for children 9–23 months) (De Vita et al., 2019; Raymond et al., 2017).

Poor feeding practices with low dietary diversity were inferred by Du Plessis et al. (2018) as contributing to high prevalence of stunting in Breede Valley Sub-district, South Africa, where about a third of children below 2 years were reported to be stunted. In Nigeria, breastfeeding beyond 1 year increased stunting risks in children, which was attributed to complementary feeding timing and quality, and mother’s educational and socioeconomic levels (Akombi et al., 2017).

A cross-sectional study, using 400 children aged 6–23 months in under-resourced communities in Tanzania, showed that locally available foods can provide infants and toddlers with nutrient requirements to prevent malnutrition (Raymond et al., 2017). Three interventions were recommended: use nutrient-dense foods to supplement local foods, increase production of those nutrient-dense foods and encourage their incorporation into regular diets of infants and toddlers so they will have the needed micronutrients (Raymond et al., 2017). Similarly, Picolo et al. (2018) and Van der Kam et al. (2016), recommended integrating micronutrient powders into infant and young child feeding programming.

### Managing healthy nutrition and lifestyle choices in pregnancy

Eleven of nineteen articles recommended that nutrition and lifestyle choices in pregnancy should be managed as they can affect foetal and child growth and development. Low body mass index before pregnancy, smoking, alcohol abuse, lack of good antenatal care, excessive physical activity and stress can all affect the intrauterine environment and ultimately the unborn child and should be addressed in relevant interventions (Carducci and Bhutta, 2018; De Vita et al., 2019).

Quelhas et al. (2018), in a systematic review and meta-analysis (210 and 124 articles, respectively), concluded that there is a negative dose-response association between tobacco use in pregnancy and baby’s anthropometric measurements (weight, length and head circumference) at birth, concluding that tobacco use in pregnancy should be discouraged. In addition to tobacco use, Britto et al. (2017), mentioned alcohol consumption in pregnancy as being detrimental to both mothers and their unborn children’s health and recommended the use of psychosocial programmes, found to be successful in 86 randomised control trials. Britto et al. (2017), however, lamented paucity of such programmes in low-to-middle-income countries. Du Plessis et al. (2018) applauded a non-governmental agency’s initiative that was mobilising communities against alcohol consumption during pregnancy thereby reducing long-term effects of foetal alcohol spectrum disorder on families.

Limited nutritional knowledge associated with a lack of formal education of child caretakers (both male and female) was found to be a problem in cross-sectional studies in Ethiopia (Mohammed et al., 2019; Zerfu and Biadgilign, 2018) and Nigeria (Akombi et al., 2017). Mothers with low body mass index – 18.5 kg/m^2^ or less – are more likely to have stunted children (Akombi et al., 2017). Mother’s nutritional statuses affect children from conception up to 6 months (Akombi et al., 2017).

Pregnant women’s nutritional practices could be addressed by strengthening existing public health and nutrition efforts. For example, at antenatal clinics, pregnant women can be educated regarding nutritional practices that improve their unborn children’s growth whenever possible (Agho et al., 2019; Bhutta et al., 2017; Mohammed et al., 2019; Shankar et al., 2017). Access to newspapers and other forms of media is encouraged in education regarding nutritional and lifestyle choices as these seem to reduce children’s chances of becoming stunted (Akombi et al., 2017), probably because of awareness leading to change in behaviour.

### Improving personal and environmental health practices

Eight of nineteen articles reported on improving personal and environmental health practices as necessary when preventing and addressing malnutrition in under-resourced communities. Poor WASH conditions were associated with stunting. Improving WASH conditions may be a public health intervention for preventing and reducing malnutrition in children, as part of the nurturing and care needed for young children to reach their full potential (De Vita et al., 2019; Richter et al., 2017).

Carducci and Bhutta (2018) discussed infections which could be fatal but easily preventable using interventions related to hygiene practices, such as found in a study conducted in a rural province in Mozambique that concluded that handwashing by mothers with a cleaning agent (soap or ashes) reduced chances of children becoming malnourished by 40% (Rose et al., 2017).

In 2017, researchers in Nigeria stated that efforts to prevent stunting in children should include interventions aimed at improving sanitation at household levels (Akombi, 2017). Two years later, Agho et al. (2019) endorsed this view by recommending that interventions aimed at improving water access and good sanitation at household levels will ultimately reduce childhood malnutrition in under-resourced communities as diarrhoea and acute respiratory infections’ incidence drops.

The correlation between acute infections and childhood malnutrition in under-resourced communities was highlighted, leading to recommendations for communities to implement effective disease control (De Vita et al., 2019). Causes of malnutrition are multi-factorial and include non-nutrition related factors that require WASH and integrated public health interventions to prevent them (Mohammed et al., 2019). Lancet’s second series of ‘Advancing Early Childhood Development’ saw WASH as an intervention needed throughout life, and advocated for access to clean water, instilling hygiene behaviours, building sanitation infrastructure and actively preventing infections in communities as part of nurturing care for childhood development (Britto et al., 2017).

### Reducing low birthweight incidence

Seven out of nineteen articles recommended that reducing low-birthweight incidences – whether due to prematurity or intrauterine growth retardation, will help prevent malnutrition within the First 1000 Days of Life. Studies in African contexts, including Ethiopia, Kenya and Nigeria, recognised that being low-birth weight increases stunting risks in children when compared to those with a birth weight above 2.5 kg, supporting recommendations that efforts must be strengthened during pregnancy to improve maternal health and nutrition to reduce low-birth weight incidence (Akombi et al., 2017; De Vita et al., 2019; Mohammed et al., 2019; Raymond et al., 2017).

A review of 86 randomised controlled trials revealed that psychosocial interventions to decrease antenatal tobacco use, when successful, reduced low-birth weight and preterm deliveries (Britto et al., 2017). This conclusion was supported by Quelhas et al. (2018), supporting inclusion of interventions to prevent prenatal exposure to tobacco use when formulating policies to reduce low-birth weight.

Lancet second series made five recommendations to improve child survival during pregnancy, delivery and postnatally. Of these, four were aimed at preventing low-birth weight, if possible, or improving chances of survival for low-birth weight babies (Britto et al., 2017). Carducci and Bhutta (2018) advocate that efforts to prevent low birth weight should include reducing teenage pregnancies, increasing intervals between pregnancies, as well as identifying and managing infections and other diseases and preventing malnutrition in pregnant women.

## Discussion

This review summarised existing best evidence practice for preventing malnutrition within the First 1000 Days of Life in under-resourced communities. After synthesising the included articles, five themes emerged, related to: improving social determinants of health using a multisector approach; enhancing infant and toddler feeding; managing healthy nutrition and lifestyle choices in pregnancy; improving personal and environmental health practices; as well as reducing low-birthweight incidence. Improving social determinants of health emerged was an intervention most frequently indicated for preventing malnutrition within the First 1000 Days of Life in under-resourced communities – evidence upheld by Heidkamp et al. (2021). Effective improvement of social determinants of health needs collaboration of different sectors (health, water, sanitation, housing, education, transport, governance structures, nutrition, social protection, safety and security) to support families to nurture and care for their children (Britto et al., 2017; Mahumud et al., 2020).

Other themes identified in this review can also be described as part of improving social determinants of health. Health inequities arise from unequal distribution of income, goods and services, which together affect the complete state of living and impact it has on nutrition, from birth to old age (WHO, 2015). This affirms what Dever (1976) portrayed in his epidemiological model for health policy analysis, which was the larger study’s theoretical framework. Dever’s model suggests that biological make-up of healthcare beneficiaries, their lifestyles and environment in which they live and work, should all be considered when health policies are being formulated as they impact health – including nutritional status – of the individual (Dever, 1976).

Interestingly, using social determinants of health approach requires some form of multi-sectoral, multidisciplinary approach. It therefore needs leadership that can mobilise stakeholders, be a mediator and manage relationships across multiple sectors as resources are leveraged to achieve a common goal (Health Policy Project, 2014).

Most articles recommended a form of multisector approach to addressing malnutrition. Using a multisector approach encourages optimisation of resources as duplications will be avoided (Salunke and Lal, 2017). Harnessing of expertise and resources of different stakeholders can be very efficient and cost-effective. Although power disparities, different organisational cultures and incompatible foci make multisectoral approaches challenging, they have been used successfully to improve health programme outcomes in other health areas (Efevbera et al., 2020). In Nepal, workshop participants were convinced that having multi-sector stakeholders address social determinants of health challenges, based on local evidence, can rouse political interest and support, which is needed for types of sustainable interventions necessary to bridge barriers with a view to improving food security and prevent malnutrition (Gaihre et al., 2019).

Only one article discussed formulating a multisector team in detail and acknowledged possible challenges but suggested they could be circumvented by using a “people-centred” approach (Du Plessis et al., 2018). ‘People-centredness’, or people-centred care, recommends that users of healthcare systems, and not disease conditions such as malnutrition, be at the centre of health decision-making and that beneficiaries – in this case caregivers and ultimately (unborn) children – should be empowered to engage in improving their health (Anderson et al., 2019; WHO, 2018).

Although, based on the definition of people-centredness, health decision-making should have beneficiaries as major stakeholders in any healthcare system decision-making, the multi-sectoral approach tends to leave these beneficiaries out of decision-making. Implementation of people-centredness requires healthcare workers to integrate services and provide continuity and coordination of care, involving stakeholders needed for holistic care of patients, including family and communities (WHO, 2018).

Care of adolescents in order to prevent malnutrition should include contraception to delay pregnancy, nutrition education, instilling hygienic habits and continued formal education to improve future work opportunities. Arlinghaus et al. (2018) support this approach though instilling hygienic habits but recommend utilising a multi-sectoral approach to reduce poverty and prevent intergenerational malnutrition.

Pregnant women should have access to quality antenatal care that will reduce occurrences of LBW by the prevention of infections and maternal conditions likely to lead to LBW delivery, such as maternal malnutrition and hypertensive diseases of pregnancy (Tunçalp et al., 2017). Alcohol and tobacco use in pregnancy may also affect foetus development and should be discouraged during antenatal care (WHO, 2016).

Feeding children starts with optimal maternal nutrition in pregnancy and breastfeeding as food of choice for the first 6 months, after which breastfeeding should be continued alongside introduction of appropriate complementary food. Researchers acknowledge that mothers who breastfeed exclusively for the first 6 months have successfully overcome barriers towards breastfeeding and have indirectly prevented malnutrition (Jama et al., 2017; Rujumba et al., 2020; Zhou et al., 2020). Barriers may be attributed to mother or baby, health system, family pressure and mothers’ employment/school-going status (Khatun et al., 2018).

Sub-optimal breastfeeding and inappropriate complementary feeding may result in malnutrition, especially if there is poor adherence to personal and environmental hygiene at individual and/or household level as chances of infections and illnesses increase (Zhou et al., 2020:9). Breastfeeding education, family support, health system and supportive legislative environment are considered enablers of breastfeeding, especially if mothers know of the benefits of breastfeeding and are determined to breastfeed (Bouras et al., 2013; Gallegos et al., 2018; Kornides and Kitsantas, 2013).

### Limitations

This review was limited to articles in English only as this was the language the authors were proficient in and, as the study was self-funded, the budget did not allow for additional translation costs. Most included articles were from African countries, despite that under-resourced communities are found worldwide. Articles, published in languages other than English, conducted on the topic in under-resourced communities outside Africa may have been missed, especially since this review only used databases the University is subscribed to, limiting accessibility to relevant articles.

### Implications for practice

This review summarised existing best evidence for preventing malnutrition within the First 1000 Days of Life in under-resourced communities. It is becoming increasingly evident that the First 1000 Days of Life presents an opportunity to improve chances of children to reach their full developmental potential. Sectors working with mothers and children, including unborn children, need to use multisectoral ‘people-centred’ approaches so beneficiaries can be empowered to participate in preventing malnutrition in the First 1000 Days of Life within under-resourced communities.

Further exploration of malnutrition dynamics in the First 1000 Days is critical. There is a specific need for high-quality studies on this topic to be done in under-resourced communities.

Findings of the present study could be used for strategy, guideline, intervention or policy-development that promote a multi-centred, people-centred approach that sets out to encompass and align all five themes identified in this review, namely: improving social determinants of health, enhancing infant and toddler feeding, managing nutrition and lifestyle choices in pregnancy, improving personal and environmental health practices and reducing low-birthweight incidence to prevent malnutrition in the First 1000 Days of Life in under-resourced communities globally, enhancing maternal and infant outcomes within this context.

## Conclusion

This review found that prevention of malnutrition within the First 1000 Days of Life in under-resourced communities requires a multisector, people-centred approach with a view to improving social determinants of health, enhancing infant and toddler feeding, managing healthy nutrition and lifestyle choices in pregnancy, improving personal and environmental health practices and reducing low-birthweight incidence. Further exploration regarding preventing malnutrition in the First 1000 Days in under-resourced communities is required using high-quality studies.

## Supplemental Material

Supplemental Material - Preventing malnutrition within the first 1000 days of life in under-resourced communities: An integrative literature review Supplemental Material for Preventing malnutrition within the first 1000 days of life in under-resourced communities: An integrative literature review by Marian J Nyarko, Dalena R M van Rooyen and Wilma ten Ham-Baloyi in Journal of Child Health Care
