# Supplementary material for: Preventing malnutrition within the first 1000 days of life in under-resourced communities: An integrative literature review
Source: J Child Health Care. 2023 Apr 3;28(4):898–913. doi: 10.1177/13674935231166427 (PMC11607848; doi:10.1177/13674935231166427)
Supplement: Supplemental Material - Preventing malnutrition within the first 1000 days of life in under-resourced communities: An integrative literature review [file sj-pdf-1-chc-10.1177_13674935231166427.pdf]

Table: Data extraction (n=13)

Reference	Design and sample	Findings	Recommendations relevant to address malnutrition in First 1000 Days of Life
**LEVEL I EVIDENCE (n=1)**			
Quelhas et al. (2018)<br>Setting/country: not applicable	Meta-analysis of Randomised Control Trials (RCTs) & Systematic review including 210 studies (systematic review), and 124 (meta-analysis)	Active tobacco use during pregnancy was associated with significantly higher rates of small-for-gestational age (pooled adjusted odds ratio [AORs]□=□1.95; 95% confidence interval [CI]: 1.76, 2.16), shorter length (pooled weighted mean difference [WMD]□=□0.43; 95% CI: 0.41, 0.44), and smaller head circumference (pooled WMD□=□0.27; 95% CI: 0.25, 0.29) at birth.	• Prenatal exposure to tobacco may lead to stunting and compromise brain development during early childhood and beyond. This should be considered when formulating policies to address child growth and development
**LEVEL II EVIDENCE (n=1)**			
Rose et al. (2015)<br>Setting/country: Mozambique	Quasi-experimental of 1472 children aged 6-59 months	Of children under age five years, 43% were undernourished in 2010 and 55% in 2014. Vitamin A supplementation was associated with a 31% (p=0.04) decreased odds of stunting. Children who were exclusively breastfed for at least six months had an 80% (p=0.02) lower odds of wasting in 2014 and 57% (p=0.05)	• Future interventions should focus on health education and economic interventions that will continue to increase the use of cleaning agents for hand washing

		decreased odds of being underweight in 2014. Introducing other foods after age six months was associated with a five-fold increased odds of wasting in 2014 (p=0.02); household food insecurity was associated with wasting (OR=2.08; p=0.03) and underweight in 2010 (OR=2.31; p=0.05). Children whose mother washed her hands with a cleaning agent had a 40% (p=0.05) decreased odds of being underweight. Per point increase in household dietary diversity score, children had 12% greater odds of being stunted in 2010 (p=0.01) but 9% decreased odds of being underweight in 2014 (p=0.02).	• Increase efforts to decrease household food insecurity<br>• Increase rates of vitamin A supplementation and vaccine uptake
**LEVEL III EVIDENCE (n=8)**			
Agho et al. (2019)<br>Country/setting: Rwanda, Tanzania and Uganda	Cross-sectional survey including 9270 children aged 0–59□months	The odds of a child being stunted were higher in Gicumbi District in Rwanda (AOR=1.00) while the odds of a child being wasted and underweight were higher in Kitgum District in Uganda (AOR=3.90; p=<□0.001). Having diarrhoea two weeks prior to the survey was significantly associated with severe undernutrition (AOR=3.66; p=<□0.001). Wealth index (least poor household), increasing child's age, sex of the child (male) and unavailability of water all year were reported to be associated with moderate or severe stunting/wasting (p=<0.001). Children of women who did not attend monthly	• Encourage monthly child growth monitoring sessions as it decreases risk of under-nutrition in those children<br>• Efforts to improve the sanitation of poor households is needed to reduce diarrhoea incidence, especially making water available throughout the year

		child growth monitoring sessions (AOR=2.19; p=<0.001) and children who had Acute Respiratory Infection symptoms were significantly associated with moderate (AOR=1.87; p=<0.001) or severe underweight (AOR=1.87; p=0.006).	• Childhood infections reduce the appetite of sufferers and can result in poor immune system and malnutrition
Akombi et al. (2017)<br>Country/setting: Nigeria	Cross-sectional study including 24,529 children aged 0–59 months	The prevalence of stunting and severe stunting were 29% [95% Confidence interval (CI): 27.4, 30.8] and 16.4% [95%CI: 15.1, 17.8], respectively for children aged 0–23 months, and 36.7% [95%CI: 35.1, 38.3] and 21% [95%CI: 19.7, 22.4], respectively for children aged 0–59 months. Multivariate analysis revealed that the most consistent significant risk factors for stunting and severe stunting among children aged 0–23 months and 0–59 months are: sex of child (male), mother's perceived birth size (small and average), household wealth index (poor and poorest households), duration of breastfeeding (more than 12 months), geopolitical zone (North East, North West, North Central) and children who were reported to having had diarrhoea in the 2 weeks prior to the survey [Adjusted odds ratio (AOR) for stunted children 0–23 months □=□1.22 (95%CI: 0.99, 1.49)];[AOR for stunted children 0–59 months□=□1.31 (95%CI: 1.16, 1.49)], [AOR for severely stunted children 0–23 months□=□1.31 (95%CI: 1.03,	• Improving maternal nutrition should reduce the incidence of low-birth-weight babies.<br>• Improving household hygiene will help reduce diarrhoeal diseases.<br>• Promoting appropriate complementary feeding<br>• Use cash transfers to improve the economic conditions for households

		1.67)]; [AOR for severely stunted children 0–59 months□=□1.58 (95%CI: 1.38, 1.82)].	
De Vita et al. (2019)<br>Country/setting: Kenya	Case Control study including 1119 babies registered at birth	The prevalence of malnutrition was high, with 26.3% of the children being stunted, 6.3% wasted and 13.16% underweight. Wasted infants were significantly associated with common childhood illnesses: with cough (OR□=□0.28 95% CI□=□0.86,0.90; p-value <0.05) and rapid breathing (OR□=□2.13; 95% CI□=□1.08, 4.22; p-value□=□0.03); as well as with diarrhea (OR□=□2.43; 95% CI□=□1.25,4.7; p-value< 0.05). Stunting was associated with hygienic conditions in households that did not perform any water treatment and for children that had a toilet within the house compound (p-value<□0.05; OR□=□0.62), immunization program (p-value<□0.05; OR□=□0.70; 95% CI□=□0.57, 0.85), and low-birth-weight (p-value<□0.05; OR 2.43; 95% CI□=□1.77, 3.32).	• Promote hygienic living, with communities having access to potable water and to toilets<br>• Plan the distribution and utilization of available healthcare resources<br>• Develop effective disease control measures through promotion of health and prevention of disease<br>• Immunization<br>• Reduce low-birth-weight infant feeding practices
Du Plessis et al. (2018)<br>Setting/country: South Africa	Non-experimental<br>Qualitative using focus group discussions and interviews with 19 stakeholders	Participants viewed knowledge and evidence about the first 1000 days of life as important to address infant and young child nutrition (IYCN). The impact of early, optimal nutrition on health and intellectual development resonated with them. Participants felt that capacity and resources were constrained by many competing agendas spreading	• Formulation of a well-planned communication and advocacy strategy to educate the community on infant and young child nutrition, with a specific

	(community members and government representatives)	public resources thinly, leaving limited scope for promotion and prevention activities. "People" were viewed as a resource, and building partnerships and relationships, could bridge some shortfalls in capacity. Conversations about politics and governance elicited strong opinions about what should be done through direct intervention, policy formulation and legislation. Participants proposed the IYCN agenda should be referred to a local, informal, inter-governmental body where directors and senior managers meet to address issues of cross-cutting importance.	focus on the First 1000 Days, of Life is needed<br>• The proposed strategy should borrow from experience gained from the HIV/AIDS campaigns
Mohammed et al. (2019)<br>Country/setting: Ethiopia	Cross-sectional survey including 2902 children aged 6–23□months	The overall prevalence of CAS was 23.9%. The dietary factors found significantly linked to lower odds of CAS were use of vitamin A supplement [adjusted odds ratio (AOR)□=□1.19, 95%CI□=□1.06–1.33, P =□0.003], consumption of vitamin A rich fruit and vegetables (AOR□=□1.15, 95%CI□=□1.04–1.27, P =□0.006), meat (AOR□=□1.55, 95%CI□=□1.17–2.05, P =□0.002), legumes (AOR□=□1.38, 95%CI□=□1.05–1.81, P =□0.021), and meal frequency□>□3 (AOR□=□1.22, 95%CI□=□1.04–1.37, P =□0.020). The non-dietary household and child factors found significantly linked to higher odds of CAS were rural residence (AOR□=□1.29,	• Strengthen existing public health and nutrition education efforts<br>• Strengthen efforts to improve infant and young child feeding practices and micronutrient supplementation<br>• Improve hygiene practices of communities<br>• Strengthen efforts to improve child health care.

		95%CI□=□1.18–1.41, P□<□0.001), low household wealth (AOR□=□1.91, 95%CI□=□1.53–2.39, P□<□0.001), low caregivers' education level (AOR□=□2.14, 95%CI□=□1.33–3.44, P□<□0.001), male sex (AOR□=□1.25, 95%CI□=□1.04–1.50, P =□0.015), age 12–23□months (AOR□=□1.65, 95%CI□=□1.57–1.73, P□<□0.001), history of infection (AOR□=□1.14, 95%CI□=□1.00–1.30, P =□0.048), and small birth size (AOR□=□1.99, 95%CI□=□1.58–2.51, P□<□0.001).	
Raymond et al. (2017)<br>Country/setting: Tanzania	Cross-sectional survey including dietary intakes of 400 children aged 6-23 months	The results showed that the prevalence of stunting, wasting and underweight for children aged 6-23 months was 30-41%, 1.5-3% and 4-9%, respectively. In addition, the results showed that diets that were consumed by the subjects comprised of local foods met vitamin A, vitamin C, protein and energy requirements for children aged 6-23 months. However, the extent of deficit in iron, zinc and calcium in baseline diets was large and difficult to meet under the existing feeding practices.	• Enrich local foods with the needed nutrients<br>• Optimise the way traditional diets are constituted to achieve the recommended nutrient intake for infants and young children
Tebeje et al. (2019)<br>Country/setting: Ethiopia	Cross-sectional survey with 7150 mothers/caretakers	About 62.55% of mothers/caretakers prefer to feed children with a family and 16.45% of them prefer to feed children with a specific type of food. Mothers/caretakers who introduce semisolid food after 6 months 2.34(1.50–	• Importance of dietary diversity must form part of the counselling given to mothers with children at

		3.96) were times more likely prefer to feed with family food for their children than a balanced diet. Regarding the specific type of food preference mothers who introduce semisolid food after 6 months and those obtain food from the market were 6.53(3.80–11.24) and 4.38(3.45–5.56) times more likely to prefer to feed specific types of than balanced diet respectively.	every visit to the clinic, including antenatal visits
Zerfu & Biadgilign (2018)<br>Country/setting: Ethiopia	Cross-sectional multi-centre survey including 389 pregnant women	Vegetables were listed top as major sources of vitamin A (45.5%) and iron (23.8%). Nearly half (47%) of the mothers lacked awareness on balanced and diversified diets. Conversely, nearly three fourths (73.8%) and two thirds (66.8%) of them had favorable attitudes towards dietary diversity and early initiation of antenatal care follow up. With a median dietary diversity score of four, starchy staples (100%), legumes and nuts (89.2%) were major food groups consumed by almost all of the mothers included in the study.	• Use antenatal clinics to improve the knowledge of mothers on maternal nutrition
**LEVEL IV EVIDENCE (n=3)**			
Britto et al. (2017)<br>Country/setting: Review of	Non-research Internationally recognized expert opinion based on	A comprehensive updated analysis of early childhood development interventions across the five sectors of health, nutrition, education, child protection, and social protection were developed.	• Pregnancy: antenatal corticosteroids and MgSO4 for women at risk of preterm birth

literature across countries (Low and middle income as well as high-income countries)	overview of systematic reviews		• At birth: Early initiation of breastfeeding, Kangaroo Mother Care (KMC)<br>• Supplementary feeding for disadvantaged children and multiple micronutrient supplementation in children<br>• Optimal infant and young child feeding<br>• Conditional cash transfers
Carducci & Bhutta (2018)<br>Country/setting: Not applicable	Non-research<br>Clinical practice guideline	With the first 1,000 days of life proving to be a critical window of opportunity for physical and cognitive growth and development, an optimal intrauterine environment is vital. If fetus needs are compromised prenatally, there is an increased risk of intrauterine growth restriction (IUGR), and infants being born premature, low birth weight (LBW), or small-for-gestational age (SGA). Specialized care of these high-risk infants is necessary in terms of preconception interventions, resuscitation, thermoregulation, nutritional support and kangaroo mother care. Significant evidence supports exclusive breastfeeding as the standard of care for feeding SGA, preterm, LBW and very low birth weight infants. Expressed	• Prioritise antenatal and postnatal care<br>• Management of growth restriction should be preventive and promotive<br>• Increase efforts that support exclusive breastfeeding as the standard of care for feeding small for gestational age (SGA), preterm, low-birth-weight (LBW) and very LBW infants<br>• Kangaroo mother care

		milk or donor milk may also require fortification, to meet higher nutrient needs of these newborns. Future research should address the gap in the literature on specific care of term and preterm IUGR and or SGA infants, and strengthening evidence for human milk bank models and emollient care.	should be standard for stable babies weighing less than 2kg<br>• Expressed milk or donor milk may also require fortification, to meet higher nutrient needs of these new-borns
Richter et al. (2017)<br>Country/setting: Review of literature across countries (Low and middle income as well as high-income countries)	Non-research<br><br>Internationally recognised expert opinion based on overview of systematic reviews	Strong biological, psychosocial, and economic arguments exist for intervening as early as possible to promote, protect, and support children's development, specifically during pregnancy and the first 2–3 years. An emphasis on the first years of life is articulated within a life course perspective that also requires quality provisions at older ages, especially during child day care and preschool, following on through schooling and into adolescence so as to capitalise on dynamic complementarities between investments made during successive lifecycle stages. Health services are particularly well placed to reach children early with services that support families to deliver nurturing care and facilitate early childhood development. A multi-sectoral framework to promote the development of young children across the life course is needed.	• Health and nutrition services are ideal starting points to scale up interventions for early childhood development<br>• Efforts to promote nurturing care of young children should be built onto existing services for maternal and child health and nutrition at no or little extra cost<br>• Health sector to look beyond disease prevention and treatment and include nurturing of infants and children
